# Lausannevirus Encodes a Functional Dihydrofolate Reductase Susceptible to Proguanil

**DOI:** 10.1128/AAC.02573-16

**Published:** 2017-03-24

**Authors:** L. Mueller, P. M. Hauser, F. Gauye, G. Greub

**Affiliations:** Institute of Microbiology, Lausanne University Hospital and University of Lausanne, Lausanne, Switzerland

**Keywords:** DHFR-TS, Lausannevirus, antifolate drugs, folate metabolism, proguanil

## Abstract

Lausannevirus belongs to the family Marseilleviridae within the group of nucleocytoplasmic large DNA viruses (NCLDVs). These giant viruses exhibit unique features, including a large genome, ranging from 100 kb to 2.5 Mb and including from 150 to more than 2,500 genes, as well as the presence of genes coding for proteins involved in transcription and translation. The large majority of Lausannevirus open reading frames have unknown functions. Interestingly, a bifunctional dihydrofolate reductase-thymidylate synthase (DHFR-TS) is encoded in the Lausannevirus genome. The enzyme plays central roles in DNA precursor biosynthesis. DHFR is the pharmacological target of antifolates, such as trimethoprim, pyrimethamine, and proguanil. First, the functionality of Lausannevirus DHFR-TS was demonstrated by the successful complementation of a DHFR-deficient Saccharomyces cerevisiae strain with a plasmid expressing the heterologous gene. Additionally, using this heterologous expression system, we demonstrated the *in vitro* susceptibility of Lausannevirus DHFR-TS to proguanil and its resistance to pyrimethamine and trimethoprim. Proguanil may provide a unique and useful treatment if Lausannevirus proves to be a human pathogen. To our knowledge, this is the first time that a DHFR-TS has been described and characterized in an NCLDV.

## INTRODUCTION

**FIG 1:**
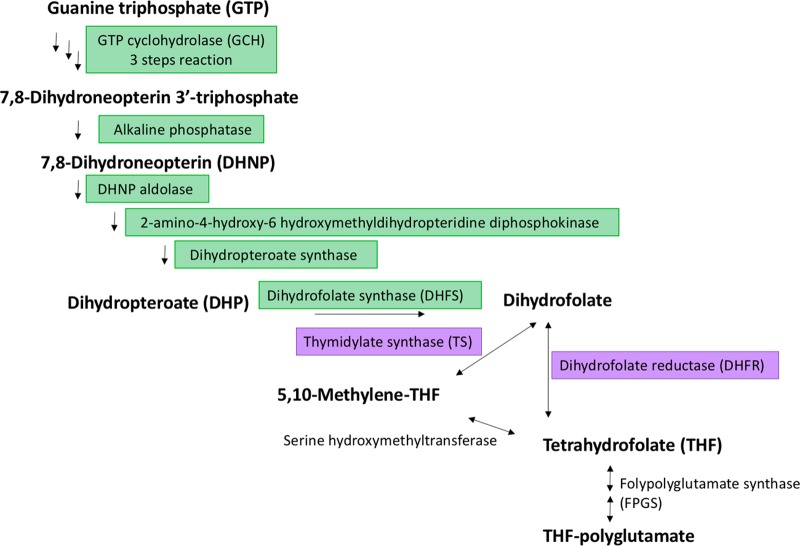
Folate biosynthesis. Shown are the main products (boldface) and enzymes involved in folate metabolism. The enzymes present only in microbes and plants are shaded in green; the enzymes detected in the Lausannevirus genome are shaded in violet. (Adapted from Kegg pathway, http://www.genome.jp/dbget-bin/www_bget?map00790.)

The giant virus Lausannevirus is part of the family Marseilleviridae, among the recently discovered group of nucleocytoplasmic large DNA viruses (NCLDVs). Comparative genomic analysis (reciprocal BLASTP and annotation) showed the presence of a bifunctional dihydrofolate reductase-thymidylate synthase (DHFR-TS)-encoding gene among the 450 predicted open reading frames (ORFs) of the 346.75-kb genome of Lausannevirus. The enzyme is involved in folate metabolism (Fig. 1). Folate is essential in all living organisms. Some of them, such as microbes and plants, are able to synthesize folate themselves, while others, such as mammals, require the uptake of folate from their diet (1, 2). More precisely, DHFR catalyzes the reduction of dihydrofolate to tetrahydrofolate, a precursor of different cofactors involved in the synthesis of several essential metabolites, such as the *de novo* synthesis of purines (3). TS is involved in *de novo* thymidylate (dTMP) biosynthesis from tetrahydrofolate. Therefore, DHFR and TS are essential enzymes for DNA synthesis and are targeted by several antimicrobial drugs. Both enzymes are expressed ubiquitously in prokaryotic and eukaryotic cells (3, 4). However, they are expressed in protozoa and plants from a single bifunctional gene, comprising the DHFR domain fused to the TS domain, while in other organisms, including humans, DHFR and TS are encoded by separate genes (5).

Several studies on DHFR and TS have been conducted in vertebrates, bacteria, and protozoa (3, 5–7). However, the enzymes have been poorly characterized in viruses. The presence of DHFR- and TS-encoding genes has been reported in T-even and T5 bacteriophages, as well as in herpesvirus saimiri (HVS) (8), herpesvirus ateles (HVA) (8), and human herpesvirus 8 (9). Varicella-zoster virus possesses its own TS gene (8). NCLDVs, such as Marseilleviridae, as well as Mimiviridae and Phycodnaviridae members, encode their own DHFR-TS proteins. A DHFR-encoding gene is also present in pandoravirus, while DHFR and TS are encoded by two separate genes in some members of the family Iridoviridae. On the other hand, a study reported that the Herpesviridae member mouse cytomegalovirus (CMV) does require the activity of the host DHFR in order to replicate in quiescent cells (10), suggesting that it does not encode its own DHFR.

Here, we demonstrate the DHFR function of the DHFR-TS protein of Lausannevirus by complementation of a Saccharomyces cerevisiae DHFR-deficient strain. Since antimicrobial agents have been reported to inhibit the DHFR activity of several prokaryotic, protozoal, and fungal parasites, including Staphylococcus aureus (11), Pneumocystis carinii (12), Plasmodium falciparum (13, 14), and Cryptosporidium parvum (12), we additionally evaluate the susceptibility of Lausannevirus DHFR-TS to trimethoprim (TM), proguanil (PG), and pyrimethamine (PYR).

## RESULTS

### DHFR and TS share conserved sites among Lausannevirus, prokaryotes, fungi, and animals.

**FIG 2:**
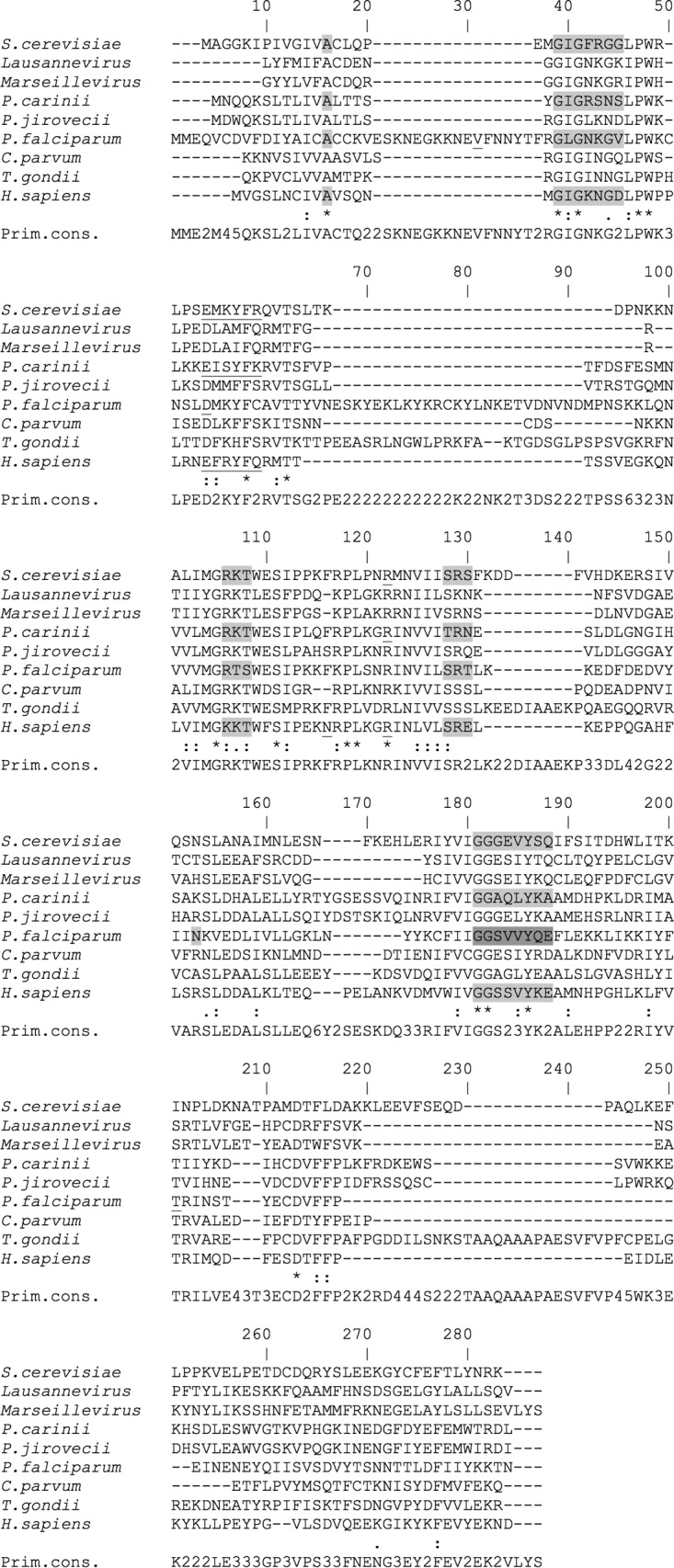
DHFR domain multiple-sequence alignment. The S. cerevisiae DHFR domain was aligned with those from other organisms. Identical amino acids are indicated by asterisks, strongly conserved amino acids by double dots, and weakly conserved amino acids by dots. Known NADP binding sites are shaded in gray. Substrate binding sites are underlined. Both NADP and substrate binding sites are indicated only in species with those annotations in Uniprot or Interpro. Note that the minor differences in the conserved sites of the DHFR sequences are mainly replacements by amino acids exhibiting similar biochemical characteristics, such as isoleucine for leucine or threonine for serine. Primary consensus (Prim. cons.) represents the calculated order of the most frequent amino acids found at each position of the sequence alignment.

DHFR conservation was assessed among several microorganisms that have been shown to successfully complement the S. cerevisiae DHFR deletant (12), as well as among those of Lausannevirus and Marseillevirus (Fig. 2). Lausannevirus and Marseillevirus DHFR domains exhibited, respectively, 22.2% and 22.8% amino acid sequence identity with S. cerevisiae DHFR. The complementing DHFRs of P. carinii, Pneumocystis jirovecii, P. falciparum, C. parvum, Toxoplasma gondii, and Homo sapiens showed 25.9%, 28.7%, 24.6%, 29.9%, 24.3%, and 27.6% amino acid sequence identity with S. cerevisiae DHFR, respectively. In summary, all the microorganisms exhibited relatively low sequence conservation compared to S. cerevisiae DHFR. However, conserved sites and conserved binding regions were identified. Similarly, the TS part of the fusion protein also showed conserved active sites, suggesting that the enzyme is likely active in Lausannevirus (see Fig. S1 in the supplemental material).

### Lausannevirus encodes a functional DHFR.

**FIG 3:**
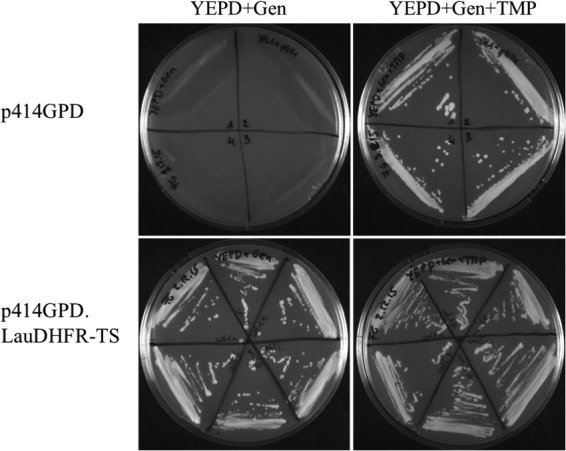
Complementation of S. cerevisiae YH1-DHFR::KanMX4 with the p414GPD.LauDHFR-TS plasmid. Transformant isolates were grown on rich YEPD medium supplemented with Geneticin in the presence or absence of dTMP. The plates were incubated at 30°C for 5 days. Only clones containing the p414GPD.LauDHFR-TS plasmid grew in the absence of TMP.

Lausannevirus DHFR-TS was successfully amplified and sequenced before being cloned into the yeast plasmid p414GPD. The constructed p414GPD.LauDHFR-TS and the empty p414GPD plasmids were introduced into the haploid S. cerevisiae strain YH1-DHFR::KanMX4, which has its own DHFR inactivated and is Geneticin resistant. Complemented strains were grown on yeast extract-peptone-dextrose (YEPD) medium supplemented with Geneticin in the presence or absence of dTMP. The six clones containing the p414GPD.LauDHFR-TS plasmid grew on the two media, whereas growth of the four clones harboring the empty p414 plasmid occurred only in the presence of dTMP (TMP) (Fig. 3). This demonstrated that Lausannevirus DHFR-TS successfully complemented the S. cerevisiae DHFR-deficient strain.

### Lausannevirus DHFR is trimethoprim and pyrimethamine resistant but susceptible to proguanil.

The susceptibility of Lausannevirus DHFR-TS to three different drugs was evaluated using the S. cerevisiae strain YH1-DHFR::KanMX4 complemented with p414GPD.LauDHFR-TS.

**FIG 4:**
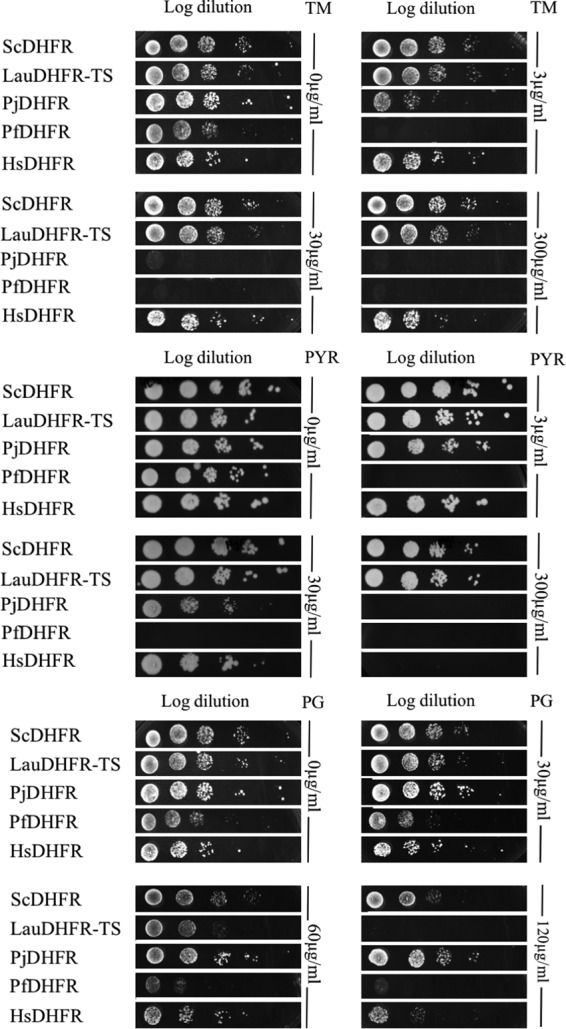
Trimethoprim, pyrimethamine, and proguanil susceptibility test. Serial dilution at 1.5 × 10^7^ cells/ml of S. cerevisiae YH1-DHFR::KanMX4 cultures complemented with S. cerevisiae DHFR (ScDHFR), Lausannevirus DHFR-TS (LauDHFR-TS), P. jirovecii DHFR (PjDHFR), P. falciparum DHFR (PfDHFR), or H. sapiens DHFR (HsDHFR) were spotted on rich YEPD medium containing 1 mM SIA and various concentrations of TM, PYR, or PG. The plates were incubated at 30°C for 3 days.

The same strain complemented with its own DHFR was used as a control, while the H. sapiens DHFR construct was used as a control for antimicrobial human toxicity. As P. jirovecii DHFR has been suggested to be trimethoprim and pyrimethamine susceptible (7) while P. falciparum DHFR is susceptible to pyrimethamine, proguanil (14), and trimethoprim (15), they were used as positive controls (Fig. 4).

The results showed that the S. cerevisiae strain expressing the heterologous Lausannevirus DHFR-TS successfully grows in the presence of low (3-μg/ml) and high (300-μg/ml) concentrations of trimethoprim or pyrimethamine. In contrast, the same strain showed reduced growth or no growth at all in the presence of proguanil at 60 and 120 μg/ml, respectively, suggesting that Lausannevirus DHFR-TS is susceptible to proguanil (Fig. 4). Reduced growth of the strain complemented with human DHFR was observed only at a high concentration of proguanil (120 μg/ml) or pyrimethamine (300 μg/ml).

## DISCUSSION

This work showed the successful complementation of the S. cerevisiae DHFR deletant by expression of the Lausannevirus DHFR-TS gene carried on a plasmid, demonstrating the functionality of the DHFR motif of the DHFR-TS gene of the virus. This procedure could improve the understanding of the functions of specific genes belonging to the poorly characterized family Marseilleviridae. Yeast complementation assays have been widely used to characterize fungal and protozoal enzymes involved in the folate pathway (12, 16, 17). Hume et al. (18) demonstrated by yeast complementation that the UL97 protein of human cytomegalovirus (HCMV) is a functional ortholog of cellular cyclin–cyclin-dependent kinase (CDK) complexes. Our study is one of the first to characterize a viral protein using this approach.

Double-stranded DNA viruses belonging to the family Herpesviridae have been shown to encode a DHFR homolog. To our knowledge, this is the first time that the DHFR activity of a bifunctional DHFR-TS has been characterized in an NCLDV. From an evolutionary perspective, it is interesting that a bifunctional fusion protein retained its DHFR activity, and this suggests that other bifunctional fusion proteins encoded by giant viruses, such as histone-like proteins (19), might also be functional. DHFR is the only member of the folate biosynthesis pathway found in the Lausannevirus genome, suggesting that the giant virus may take the dihydrofolate needed for its replication from its hosts. Consistently, Acanthamoeba castellanii, from which Lausannevirus was isolated (19), bears coding sequences for the majority of the enzymes involved in folate biosynthesis (20). In contrast, the Herpesviridae member HCMV was shown to induce the activation of host DHFR transcription (21), which might help to ensure a sufficient supply of deoxynucleoside triphosphates (dNTPs), necessary for virus replication.

We demonstrated the *in vitro* susceptibility of the Lausannevirus DHFR motif of the DHFR-TS-encoding gene to proguanil and its resistance to pyrimethamine and trimethoprim. On the other hand, proguanil inhibited human DHFR only at high concentrations. Such a result is promising for the development of treatments targeting the Lausannevirus DHFR-TS-encoding gene. So far, Lausannevirus and other members of the family Marseilleviridae have not been proven to be human pathogens. However, human exposure to these viruses has been demonstrated by several studies (22–27). Notably, Senegalvirus has been isolated from human stool (27), and Marseillevirus was detected in lymph nodes of various patients (24–26).

Marseilleviridae DHFR-TS protein showed high sequence conservation. The amino acid sequence identity with Lausannevirus DHFR-TS (YP_004347389.1) was 62% for Marseillevirus (YP_003407153.1), Melbournevirus (YP_009094883.1), and Cannes 8 virus (AGV01804.1) but 80% for Brazilian Marseillevirus (YP_009238985.1) and 76% for golden mussel Marseillevirus (YP_009310371.1). Thus, proguanil might also be effective on other members of the Marseilleviridae. It is therefore of major interest to study and characterize these viruses and potential new drugs. Proguanil might provide a treatment if Lausannevirus and/or other members of the Marseilleviridae prove to be human pathogens. Nevertheless, additional studies are needed to confirm its effect on the multiplication of other Marseilleviridae members.

## MATERIALS AND METHODS

### DHFR sequence alignment.

DHFR protein sequences were aligned using ClustalW embedded in Network Protein Sequence Analysis (NPS@) version 3.0 (28, 29). UniProt (30) and InterPro (31) were used to find conserved active and binding sites. The homology of all proteins involved in the folate biosynthesis pathway (Fig. 1) to that of Lausannevirus was investigated by BLASTX (32). Further, the absence of a specific domain signature of folate biosynthesis in the proteins surrounding DHFR was verified by Interpro. Amino acid sequence identities were assessed by BLASTP (32). The sequence accession numbers of the DHFR and DHDF-TS proteins aligned are as follows: S. cerevisiae, NP_014879.1; Lausannevirus, YP_004347389.1; Marseillevirus, YP_003407153.1; P. carinii, AAA33787.1; P. jirovecii, AAF14071.1; P. falciparum, XP_001351479.1; C. parvum, AAC47229.1; T. gondii, XP_002367252.1; H. sapiens, AAA58485.1. For alignment of the DHFR domains, DHFR-TS-encoding genes were truncated at the following amino acid positions: Lausannevirus, 171; Mareillevirus, 174; C. parvum, 178; P. falciparum, 230; and T. gondii, 251.

### Lausannevirus purification and DNA extraction.

Lausannevirus was purified as described previously by Thomas et al. (19). Briefly, Lausannevirus was cocultured with A. castellanii ATCC 30010 in 30 ml of peptone-yeast extract-glucose (PYG) medium (33) within 75-cm^2^-surface cell culture flasks (Becton Dickinson). The flasks were kept at 32°C until complete amoebal lysis was observed (usually between 24 and 48 h); then, cocultures were harvested and centrifuged at 5,000 × *g* for 15 min. The supernatant was collected and passed through 5-μm filters to remove residual amoebal cells; then, the filtrate was centrifuged at 35,000 × *g* for 1 h, and the pellet was resuspended in 1 ml of DNA-free water. Lausannevirus DNA was further isolated from 50-μl viral suspensions according to the instructions for the Wizard SV genomic DNA purification system (Promega), using a 2-h incubation time for digestion and 50 μl of nuclease-free water for elution.

### Construction of the p414GPD.LauDHFR-TS plasmid.

Lausannevirus DHFR-TS was amplified by PCR, using the Phusion high-fidelity DNA polymerase (NEB, Allschwil, Switzerland) and the following primers: DHFR_fw (5′-ATATTGGATCCCTAAATGTCGTTATACTT-3′) and DHFR_rev (5′-CAATAGTCGACTTTTTTAGACTGCCATAG-3′), creating unique BamHI and SalI restriction sites in the PCR product (underlined in the primers). The PCR products were purified using the MSC Spin PCRapace kit (Stratec Molecular, Birkenfeld, Germany). The PCR products and the p414GPD expression vector (ATCC 87356) (34), which contains an ampicillin resistance (*ampR*) gene and a tryptophan marker (TRP1), were digested using BamH1-HF and Sal1-HF (NEB, Ipswich, MA, USA) for 15 min at 37°C. Lausannevirus DHFR-TS was ligated for 1 h at room temperature to the p414GPD plasmid at a 3:1 (insert/vector) ratio; the T4 DNA ligase and its buffer were from Promega (Dübendorf, Switzerland). Recombinant plasmids were transformed in chemically competent Escherichia coli TOP10 (Thermo Fisher Scientific, Waltham, MA, USA). Briefly, 10 μl of ligation product was added to the bacteria, which were placed for 10 min on ice. Heat shock was performed at 42°C for 45 s, and the bacteria were then put back on ice. Five hundred microliters of SOC medium (20 g/liter tryptone, 5 g/liter yeast extract, 4.8 g/liter MgSO_4_, 3.603 g/liter dextrose, 0.5 g/liter NaCl, and 0.186 g/liter KCl ) was added to each sample and incubated for 1 h at 37°C with shaking; 200 μl lysogeny broth (LB) medium (1% [wt/vol] Difco tryptone, 0.5% Difco yeast extract, 1% NaCl, 2% Gibco agar) containing ampicillin (1 μl/ml; AppliChem, St. Louis, MO, USA) was incubated at 37°C overnight. The constructions were verified by sequencing performed using BigDye Terminator, 30 ng of plasmid DNA, and 3.2 pmol of the following primers: DHFR_fw, DHFR_rev, and T7_fw (5′-TAATACGACTCACTATAGGG-3′). The products were then purified with SigmaSpin postreaction cleanup and sequenced with Sanger technology. The sequences were visualized with Geneious version R7.1.9 (35).

### Complementation of the S. cerevisiae DHFR deletant with the p414GPD.LauDHFR-TS plasmid.

The DHFR-deficient S. cerevisiae strain YH1-DHFR::KanMX4 (MAT**a** or MATα; *ura3*Δ*0 leu2*Δ*0 his3*Δ*1 met15*Δ*0 lys2*Δ*0* YOR236w::*kanMX4*) was obtained by dissection of tetrads of strain Y22492 from the European Saccharomyces cerevisiae Archive for Functional Analysis (http://www.euroscarf.de) on YEPD rich medium (1% [wt/vol] Difco yeast extract, 2% Difco peptone, 2% glucose) supplemented with dTMP at 100 μg/ml (TMP) (Sigma-Aldrich, Buchs, Switzerland). TMP supplements the absence of DHFR and is required for the growth of S. cerevisiae strain YH1-DHFR::KanMX4. The strain was transformed with the empty p414GPD plasmid, as well as with the previously constructed p414GPD.LauDHFR-TS plasmid, by the one-step method described by Chen et al. (36). Briefly, the YH1-DHFR::KanMX4 strain was grown for 3 days at 30°C and 1 day at room temperature in YEPD supplemented with TMP at 100 μg/ml and Geneticin at 100 μg/ml (Gibco, Thermo Fisher Scientific). Further, the culture was centrifuged for 10 min at 5,500 rpm, and the pellet was resuspended in 1.3 ml of polyethylene glycol (PEG)-lithium acetate (LiAc) solution (15 mg/ml dithiothreitol [DTT], 200 mM LiAc, 50% [wt/vol] PEG 8000, and 250 μg/ml salmon sperm DNA denatured for 5 min at 95°C). Then, the plasmid was added to yeast cells and incubated for 1 h at 45°C, after which the cells were spun for 5 min at 13,000 rpm and the pellet was resuspended in 100 μl NaCl (0.9%). The suspension was finally spread on yeast nitrogen base (YNB) poor medium (0.67% [wt/vol] Difco yeast nitrogen base, 2% glucose, 2% Gibco agar) supplemented with Complete supplement mixture (CSM) lacking tryptophan (MP Biomedicals), Geneticin, and dTMP, and the plates were incubated for 5 days at 30°C. To confirm the functional complementation, colonies were grown on YEPD medium supplemented with Geneticin with or without TMP.

### Evaluation of the p414GPD.LauDHFR-TS construct's susceptibility to antimicrobial agents.

The strain YH1-DHFR::KanMX4 complemented with p414GPD.LauDHFR-TS was grown in liquid YEPD medium for 2 days at 30°C. Absorbance at 540 nm was measured, and the cultures were diluted in 0.9% NaCl to obtain 1.5 × 10^7^ cells/ml. Tenfold serial dilutions were assessed before inoculating the cells into solid YEPD medium supplemented with 1 mM sulfanilamide (SIA). To test antimicrobial agents' susceptibilities, the same diluted cultures were inoculated into solid YEPD medium containing 1 mM SIA and supplemented with different concentrations (0 μg/ml, 3 μg/ml, or 300 μg/ml) of TM, PG, or PYR. The PG MIC was further assessed by adding 0 μg/ml, 30 μg/ml, 60 μg/ml, or 120 μg/ml of PG to the medium. The plates were incubated for 3 days at 30°C. As controls, the same analyses were performed with strain YH1-DHFR::KanMX4 complemented by constructs carrying the DHFR genes of other organisms. The constructs used contained the DHFR proteins from Lausannevirus (see above), S. cerevisiae (GR7.ScDHFR), H. sapiens (GR7.HsDHFR), and P. falciparum (GR7.PfDHFR) (12) (kindly provided by C. Sibley). The plasmid containing the heterologous DHFR of P. jirovecii (GR7.PjDHFR) (kindly provided by L. Ma) was described by Ma et al. (12, 37). Each experiment was repeated 3 times.

## Supplementary Material

Supplemental material
