# Supplementary material for: Lausannevirus Encodes a Functional Dihydrofolate Reductase Susceptible to Proguanil
Source: Antimicrob Agents Chemother. 2017 Mar 24;61(4):e02573-16. doi: 10.1128/AAC.02573-16 (PMC5365716; doi:10.1128/AAC.02573-16)
Supplement: Supplemental material [file supp_61_4_e02573-16__index.html]

Supplemental material 

# Lausannevirus Encodes a Functional Dihydrofolate Reductase Susceptible to Proguanil

## Supplemental material

- Supplemental file 1 -

  Supplemental Figure S1

  PDF, 45K
