# Supplementary material for: Lausannevirus Encodes a Functional Dihydrofolate Reductase Susceptible to Proguanil
Source: Antimicrob Agents Chemother. 2017 Mar 24;61(4):e02573-16. doi: 10.1128/AAC.02573-16 (PMC5365716; doi:10.1128/AAC.02573-16)
Supplement: Supplemental material [file AAC.02573-16_zac004176053s1.pdf]

SUPPLEMENTAL MATERIAL

Fig. S1| **Alignment of Thymidilate synthase (TS) domains from different organisms.**

The TS domain of Lausannevirus was aligned with those of other pathogenic organisms. Identical amino acid are shown by asterisks, strongly conserved by double dots and weakly conserved by dots. Known dUMP binding sites are highlighted in grey, active sites are underlined. Both, binding and active sites are notified only in species presenting this annotation in Uniprot or Interpro. Prim. cons., primary consensus (see legend of Figure 2).

```
                          10        20        30        40        50
                           |         |         |         |         |
Lausannevirus    ---------------------------------------------FQAAMF
Marseillevirus   ------------------KSSHN---------------------FETAMM
Pfalciparum      --DDFVYFNFNKEKEEKNKNSIHPN----------------DFQIYNSLK
Cparvum          EKKTLQNCDPVRGQLKSIDDTVDLLGEIFGIRKMGNRHKFPKEEIYNTPS
Tgondii          ---VLAWMDEEDRKKREQKELIR---------------------AVPHVH

Prim.cons.       EK23L333D3333K3333K4SI422GEIFGIRKMGNRHKFP2222YNAM5

                          60        70        80        90       100
                           |         |         |         |         |
Lausannevirus    HNS--DSGELGYLALLSQVVNYGDERQDRTGTGTKSLFAKTLHFPNVSEN
Marseillevirus   FRK--NEGELAYLSLLSEVLYSGEKRKDRTGTGTRSLFGRHLLFENISEK
Pfalciparum      YKY---HPEYQYLNIIYDIMMNGNKQSDRTGVGVLSKFGYIMKFD-LSQY
Cparvum          IRFGREHYEFQYLDLLSRVLENGAYRENRTGISTYSIFGQMMRFD-MRES
Tgondii          FRG---HEEFQYLDLIADIINNGRTMDDRTGVGVISKFGCTMRYS-LDQA
                  .      *  ** ::  ::  *    .:*** .. * *.  : :  : :
Prim.cons.       FR5GR3HGE2QYLDLLSDVLNNG5KR5DRTG2GT5S2FG5TMRFDNLSE5

                         110       120       130       140       150
                           |         |         |         |         |
Lausannevirus    FPLLTVKKTNWDKILSELLWFLSGSTDATILKEKGNDIWDGNASKEFQEK
Marseillevirus   FPLITVKRMAWGCILSELLWFLSGSTDSKMLELQNNNIWKKNSSRKFLDG
Pfalciparum      FPLLTTKKLFLRGIIEELLWFIRGETNGNTLLNKNVRIWEANGTREFLDN
Cparvum          FPLLTTKKVAIRSIFEELIWFIKGDTNGNHLIEKKVYIWSGNGSKEYLER
Tgondii          FPLLTTKRVFWKGVLEELLWFIRGDTNANHLSEKGVKIWDKNVTREFLDS
                 ***:*.*:     ::.**:**: *.*:.. *  :   **. * :::: :
Prim.cons.       FPLLTTKKV2WRGILEELLWFI2G2TN2NHL5EK2V5IWD2NGSREFLD5

                         160       170       180       190       200
                           |         |         |         |         |
Lausannevirus    VGLSHYEEGDCGPIYPFQWRHAGAKYVDCKKDYTGEGKDQILEMVRLIQE
Marseillevirus   RGLS-YREGDCGPIYGFQWRHWGAKYVDCDTDYRGQGEDQILSIISEIQN
Pfalciparum      RKLFHREVNDLGPIYGFQWRHFGAEYTNMYDNYENKGVDQLKNIINLIKN
Cparvum          IGLGHREENDLGPIYGFQWRHYNGEYKTMHDDYTGVGVDQLAKLIETLKN
Tgondii          RNLPHREVGDIGPGYGFQWRHFGAAYKDMHTDYTGQGVDQLKNVIQMLRT
                   *    . .* ** * ***** .. *     :* . * **: .::  ::
Prim.cons.       RGLSHREEGD2GPIYGFQWRHFGA2Y2DMH2DYTGQGVDQL2NII5LI2N

                         210       220       230       240       250
                           |         |         |         |         |
Lausannevirus    DPTSRRILLESWNVADLDKMVLPPCHKTFQVYVRGDK-IDGQVYQRSADL
Marseillevirus   NATSRRLVLSAWNVSDLDKMCLPPCHSFSQFYVRGDF-VDCHLYQRSADL
Pfalciparum      DPTSRRILLCAWNVKDLDQMALPPCHILCQFYVFDGK-LSCIMYQRSCDL
Cparvum          NPKDRRHILTAWNPSALSQMALPPCHVLSQYYVTNDNCLSCNLYQRSCDL
Tgondii          NPTDRRMLMTAWNPAALDEMALPPCHLLCQFYVNDQKELSCIMYQRSCDV
                 :...** :: :**   *.:* *****   * **  .   :.  :****.*:
Prim.cons.       NPTSRRILLTAWNV2DLD2MALPPCH5L2QFYVR2DK2LSCI2YQRSCDL

                         260       270       280       290       300
                           |         |         |         |         |
Lausannevirus    ALGVPFNIASYACLLSLLAKRTGKSAGNLTLCFGDVHVYKNHMENAQKML
Marseillevirus   ALGVPFNIASYACLLSIIAEASGKKAGNLTMSFGDVHIYETHIDNAQKML
Pfalciparum      GLGVPFNIASYSIFTHMIAQVCNLQPAQFIHVLGNAHVYNNHIDSLKIQL
Cparvum          GLGSPFNIASYAILTMMLAQVCGYEPGELAIFIGDAHIYENHLTQLKEQL
Tgondii          GLGVPFNIASYSLLTLMVAHVCNLKPKEFIHFMGNTHVYTNHVEALKEQL
                 .** *******: :  ::*.  . .. ::   :*:.*:* .*:   :  *
Prim.cons.       GLGVPFNIASYA2LTSM2AQVCG2KPG2L2HFFGD2HVYENHI2NLK2QL

                         310       320       330       340
                           |         |         |         |
Lausannevirus    ERVPHRPPILEIVN-IADDTLKKLEPSDFVLKDYKSYSALNFAMAV
Marseillevirus   ERLPHKTPKLKVK--ISDNTLFCLKKGDFELDGYSCCGALQFDMA-
Pfalciparum      NRIPYPFPTLKLN--PDIKNIEDFTISDFTIQNYVHHEKISMDMA-
Cparvum          SRTPRPFPQLKFK--RKVENIEDF----------------------
Tgondii          RREPRPFPIVNILNKERIKEIDDFTAEDFEVVGYVPHGRIQMEMAV
                  * *   * ::.      . :  :
Prim.cons.       ER5P2PFPILKIKNKI52K2IEDFT4SDFEL4GYV4HGA2Q2DMAV
```
